# Supplementary material for: Impact of Fluxapyroxad and Mefentrifluconazole on Microbial Succession and Metabolic Regulation in Rice Under Field Conditions
Source: Foods. 2025 May 27;14(11):1904. doi: 10.3390/foods14111904 (PMC12155283; doi:10.3390/foods14111904)
Supplement: Supplementary file 1 [file foods-14-01904-s001.zip › foods-3623923-supplementary.pdf]

Supplementary Material

Impact of Fluxapyroxad and Mefentrifluconazole on Microbial Succession and Metabolic Regulation in Rice Under Field Conditions

Changpeng Zhang [1], Nan Fang [1], Chizhou Liang [2], Xiangyun Wang [1], Yanjie Li [1], Hongmei He [1], Xueping Zhao [1], Yuqin Luo [1,*] and Jinhua Jiang [1,*]

[1] State Key Laboratory for Managing Biotic and Chemical Threats to the Quality and Safety of Agro-Products, Ministry of Agriculture and Rural Affairs Key Laboratory for Pesticide Residue Detection, Institute of Agro-Products Safety and Nutrition, Zhejiang Academy of Agricultural Sciences, Hangzhou 310021, China; cpzhang1215@zaas.ac.cn (C.Z.); fn199198@hotmail.com (N.F.); wangxiangyun2@sina.com (X.W.); yanjieli0913@outlook.com (Y.L.); hehongmei53@163.com (H.H.); zhaoxp@zaas.ac.cn (X.Z.)
[2] Plant Protection Quarantine and Pesticide Management Station of Zhejiang, Hangzhou 310020, China; czliang1975@163.com
* Correspondence: lyq13665805502@163.com (Y.L.); jiangjh@zaas.ac.cn (J.J.)

Supplementary text

1. Materials and Methods

1.1 DNA extraction methodology

CTAB Protocol: ① Tissue homogenization: the soil was ground in liquid nitrogen and transferred to a 1.5 mL tube containing 700 μL preheated (65°C) CTAB lysis buffer (2% CTAB, 100 mM Tris-HCl pH 8.0, 20 mM EDTA, 1.4 M NaCl, 1% PVP-40). ② Cell lysis: Samples were incubated at 65°C for 30 min with occasional gentle mixing. ③ Protein removal: An equal volume of chloroform:isoamyl alcohol (24:1) was added, mixed by inversion for 10 min, and centrifuged at 12,000 ×g for 15 min. The aqueous phase was transferred to a new tube. ④ DNA precipitation: Cold isopropanol (0.7 volumes) was added, mixed gently, and incubated at −20°C for 1 hr. DNA was pelleted by centrifugation (12000 ×g, 10 min). ⑤ Washing: The pellet was washed twice with 70% ethanol, air-dried, and dissolved in 50 μL TE buffer (10 mM Tris-HCl pH 8.0, 1 mM EDTA). ⑥ Quality control: DNA purity (A_{260}/A_{280}: 1.8–2.0) and concentration were measured using a NanoDrop spectrophotometer.

Endophytic and non-epiphytic microbiota: Surface sterilization: roots were treated with 3% NaClO (5 min) and 70% ethanol (2 min) to remove epiphytes. Validation: post-sterilization, surface

rinse solutions were subjected to PCR amplification (universal 16S rRNA primers) to confirm the absence of residual microbial DNA (negative controls). Endophytes: DNA was extracted from surface-sterilized root tissues, ensuring only internally colonized microbes were analyzed. Rhizosphere microbiota (non-epiphytic microbiota): DNA was extracted from rhizosphere soil (adhering to roots but not sterilized), representing microbes on root surfaces and surrounding soil. Negative controls: sterilized blank root samples were processed to validate sterilization efficacy. Positive controls: DNA from unsterilized roots confirmed the presence of epiphytes. This methodology effectively isolates endophytic (intra-root) and rhizosphere-associated (non-epiphytic) communities. Detailed protocols are provided in Supporting information.

2. Results and discussion

2.1 Optimization of cleanup process

ACN was chosen as an extractive solvent for its benefits, including higher recoveries on a broad range of pesticides and fewer co-extracted matrix components [27]. Additionally, the effects of three purification compounds (PC with 0 mg, 8 mg and 50 mg including 50 mg PSA, 50 mg C18, and 150 mg MgSO4) on the recovery yields of MFZ and FXP at fortifications of 10 μg/kg were examined. While purifying soil samples with 0 mg PC, the recoveries of FXP and MFZ were 88.88±2.65% and 86.64±1.92%; 79.84±0.41% and 77.20±2.22% using 50 mg PC to purify rice husk (Figure S1). However, with the application of 8 mg PC, the recoveries of all matrices, including brown rice, rice husk, rice root and soil, ranged from 75.59±0.53% (root) to 89.49±3.23% (soil) with minimal pigment effect. Therefore, the ultimate absorbing material used for preparing the samples was 8 mg PC + 50 mg PSA + 50 mg C18 + 150 mg MgSO4.

Supplementary table

Table S1 Linear regression parameters of calibration curve of FXP and MFZ in solvent and four matrices for 0.1~50 μg/L.

Compound	Matrix	Regression equation	R^2	Calibration range (μg/L)	LOQ (μg/kg)	Matrix effect (%ME)
FXP	solvent	y = 3540343 x + 18096	0.9995	0.1~50	10	-
	soil	y = 3222789 x +13032	0.9999	0.1~50	10	-8.97
	rice root	y = 1821479 x + 7965	0.9936	0.1~50	10	-48.55
	rice husk	y = 528091 x + 597	0.9914	0.1~50	10	**-85.08**
	brown rice	y = 1804026 x + 7904	0.9990	0.1~50	10	-49.04
MFZ	solvent	y = 2146287 x + 422	0.9999	0.1~50	10	-
	soil	y = 2023962 x - 646	0.9997	0.1~50	10	-5.70
	rice root	y = 1191766 x + 348	0.9972	0.1~50	10	-44.47
	rice husk	y = 494192 x - 566	0.9939	0.1~50	10	**-76.97**
	brown rice	y = 1208670 x - 693	0.9988	0.1~50	10	-43.69

Table S2 Residual kinetics, correlation coefficients (R^2) and half-lives ($T_{1/2}$) of MFZ and FXP during the dissipation in soil, rice root, rice husk and brown rice.

Pesticides	Molecular weight	Chemical structure	Log Kow	Matrix	Residual kinetics	R^2	$T_{1/2}$ (days)
FXP	381.30	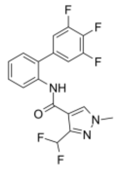	3.13	soil	$y = 0.0907e^{-0.033x}$	0.9033	21.00
				rice root	$y = 0.0065e^{0.0628x}$	-	-
				rice husk	$y = 6.1417e^{-0.064x}$	0.8898	10.83
				brown rice	$y = 0.0166e^{0.0187x}$	-	-
MFZ	397.78	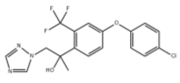	3.4	soil	$y = 0.0546e^{-0.027x}$	0.8666	25.67
				rice root	$y = 0.0054e^{0.0638x}$	-	-
				rice husk	$y = 1.5803e^{-0.03x}$	0.9302	23.10
				brown rice	$y = 0.0154e^{-0.012x}$	0.9176	57.76

Table S3 MRLs registered of FXP and MFZ in rice by various countries.

Pesticides	MRLs (mg/kg)							ADI	ARfD
	China	CAC	EU	Korea	Japan	America	Australia	(mg/kg bw/day)	(mg/kg bw)
FXP	1*	3	5	0.05	3	8	0.1	0.02	0.3
MFZ	-	5	0.01	-	4	4	-	0.04	0.3[e]

* Represents this limit is a temporary limit.

Table S4 Richness and diversity indices of rhizosphere bacterial communities under CK7D, CK21D, LF7D and LF21D treatments.

Treatments	Niches	OTUs (±SD)	Coverage (±SD)	Chao1 (±SD)	Shannon index (±SD)
CK7D	Rhizosphere	2599±783	0.99±0.003	2660.76±820.99	10.54±0.56
CK21D	Rhizosphere	2321±468	1.00±0.002	2366.74±490.51	10.45±0.31
LF7D	Rhizosphere	2218±609	1.00±0.002	2257.11±635.43	10.35±0.43
LF21D	Rhizosphere	2683±401	0.99±0.002	2744.85±431.41	10.74±0.21

Table S5 Richness and diversity indices of endosphere bacterial communities under CK7D, CK21D, LF7D and LF21D treatments.

Treatments	Niches	OTUs (±SD)	Coverage (±SD)	Chao1 (±SD)	Shannon index (±SD)
CK7D	Endosphere	1489±422	1.00±0.002	1531.37±437.84	8.14±0.68
CK21D	Endosphere	712±219	1.00±0.001	739.42±229.43	5.44±1.39
LF7D	Endosphere	993±172	1.00±0.0008	1028.70±173.16	6.62±0.82
LF21D	Endosphere	982±255	1.00±0.001	1004.97±267.54	6.93±0.73

Table S6 DEMs discriminating LF7D from CK7D.

Compound ID	m/z	Retention time (min)	Ion mode	Metabolites	kegg	VIP	P-value	ratio	regulated
HMDB0000114	215.05536	0.801	neg	sn-glycero-3-phosphoethanolamine	C01233	1.044748886	1.60157E-06	0.411971136	down
HMDB0000134	116.01057	0.807	neg	fumaric acid	C00122	2.096992282	6.77336E-05	2.036170087	up
HMDB0014850	244.08989	0.807	neg	flurbiprofen	C07013	1.185898925	3.49671E-07	0.24357604	down
HMDB0000089	243.08681	0.813	neg	cytidine	C00475	1.139478534	3.19704E-08	0.241682311	down
HMDB0000606	148.03666	0.832	neg	(r)-2-hydroxyglutarate	C01087	1.743774694	0.001753896	2.116202165	up
HMDB0011732	194.04212	0.855	neg	2-dehydro-d-gluconate	C06473	1.890075533	9.31454E-07	2.789417198	up
HMDB0000034	135.05429	0.903	neg	adenine	C00147	1.323794735	5.79681E-05	2.415297286	up
HMDB0014502	378.11586	2.264	neg	mefloquine	C07633	2.12913847	2.18737E-07	0.435619593	down
HMDB0060471	311.10033	3.267	neg	dhurrin	C05143	1.217477325	2.04156E-06	0.477620691	down
HMDB0000752	146.05752	3.314	neg	3-methylglutaric acid		1.11526772	4.31712E-05	2.26878164	up
HMDB0001895	138.03129	3.462	neg	salicylic acid	C00805	2.301185038	1.28378E-11	0.297883365	down
HMDB0001964	180.04184	3.805	neg	caffeic acid	C01197	2.671508672	1.71123E-11	0.453429658	down
HMDB0002122	398.22987	4.523	neg	prostaglandin f3	C06476	2.41926308	5.44636E-10	0.10444698	down
HMDB0000623	230.15133	4.543	neg	dodecanedioic acid	C02678	2.435442445	1.96405E-07	3.566840437	up
HMDB0000954	194.05736	4.87	neg	ferulic acid	C01494	1.70652445	1.91853E-07	0.249418703	down
HMDB0002122	398.22994	5	neg	prostaglandin f3	C06476	2.130351235	0.000406925	0.18774365	down
HMDB0039275	292.16717	5.334	neg	gingerdione	C10459	1.812949438	0.000210606	0.477103079	down
HMDB0141782	178.06252	5.374	neg	coniferylaldehyde	C02666	2.294749792	2.0882E-07	0.156182224	down
HMDB0030228	280.08421	5.982	neg	methyl nigakinone	C16996	2.388798799	0.000109803	0.083412844	down
HMDB0032489	460.28221	8.164	neg	hydrocortisone caproate	C13422	2.348500969	1.35424E-07	0.201593359	down

HMDB0000114	215.05529	0.829	pos	sn-glycero-3-phosphoethanolamine	C01233	1.102716336	7.80242E-08	0.46608508	down
HMDB0011729	504.16802	0.832	pos	1f-beta-d-fructosylsucrose	C03661	1.600319456	0.005217089	0.319368758	down
HMDB0000191	133.03715	0.839	pos	l-aspartic acid	C00049	1.812745985	0.001995719	2.110617137	up
HMDB0000086	257.10216	0.846	pos	glycerophosphocholine	C00670	1.529484945	1.32754E-09	0.29790939	down
HMDB0001046	176.09465	0.917	pos	cotinine		1.272803641	0.001922527	0.389321224	down
HMDB0014581	136.03827	0.918	pos	allopurinol		2.142835859	3.50917E-08	2.39452991	up
HMDB0004225	173.0796	0.918	pos	5-guanidino-2-oxopentanoate	C03771	1.558140511	0.001762308	0.462736395	down
HMDB0034301	85.08897	1.564	pos	piperidine	C01746	1.506276806	0.002765003	0.011168159	down
HMDB0001645	131.09434	1.565	pos	l-norleucine	C01933	1.475913805	0.004427691	0.007381712	down
HMDB0060485	236.07923	1.576	pos	l-formylkynurenine	C02700	1.734898274	0.001677637	0.040992765	down
HMDB0062189	157.05244	1.668	pos	1-nitrosonaphthalene	C14788	1.442417568	0.00841172	0.030532521	down
HMDB0000824	217.13089	3.334	pos	o-propanoylcarnitine	C03017	1.614616273	6.25309E-06	2.112095108	up
HMDB0014818	427.15866	3.49	pos	moricizine	C07743	2.65007834	1.00319E-06	0.159020796	down
HMDB0062769	113.08384	3.59	pos	caprolactam	C06593	1.373439047	5.35058E-06	0.493288462	down
HMDB0059747	251.07892	4.066	pos	n-feruloylglycine	C02564	2.375615555	1.02694E-06	2.264716929	up
HMDB0006465	568.2809	4.529	pos	ltf4	C06462	1.003421973	0.007513797	0.35046474	down
HMDB0002710	316.20308	4.563	pos	prostaglandin j2	C05957	2.506197357	7.95624E-07	0.099111595	down
HMDB0059925	180.11462	4.645	pos	2-tert-butyl-4-methoxyphenol		2.610835072	1.16061E-06	7.808983576	up
HMDB0061919	151.06292	4.659	pos	2-acetamidophenol		2.44062557	8.58289E-10	7.445713665	up
HMDB0001218	146.03647	5.409	pos	coumarin	C05851	2.525502077	8.6581E-09	0.152060628	down
HMDB0002121	330.18212	6.052	pos	carnosol	C09069	1.772220318	2.44936E-05	0.081507903	down
HMDB0002121	330.18231	6.073	pos	carnosol	C09069	1.262601001	6.74744E-05	0.141513282	down
HMDB0061861	290.18749	6.111	pos	octyl methoxycinnamate		1.35993961	1.88839E-05	3.233430514	up
HMDB0002121	330.18217	6.125	pos	carnosol	C09069	2.113790317	2.26561E-05	0.215775624	down
HMDB0001218	146.0365	6.36	pos	coumarin	C05851	1.677005475	0.000437311	2.029845548	up
HMDB0061861	290.18754	6.53	pos	octyl methoxycinnamate		1.844474271	3.9122E-06	2.499876926	up

HMDB0002725	274.19266	6.737	pos	nandrolone	C07254	2.122497688	0.000172458	2.688189242	up
HMDB0060906	330.2188	6.938	pos	11-hydroxy-delta-9-thc		2.120902332	2.97776E-06	0.09856346	down
HMDB0005096	361.2608	7.084	pos	n-arachidonylglycine		1.215387389	0.003269732	0.309108766	down
HMDB0013195	169.08872	7.113	pos	4-aminobiphenyl	C10998	1.4962698	5.21167E-05	2.09116929	up
HMDB0006031	286.19247	7.299	pos	11-oxoetiocholanolone	C14552	1.607213714	7.61701E-06	0.213296709	down
HMDB0005085	363.27672	7.304	pos	leukotriene b4 dimethylamide		1.985675098	0.0002915	0.114210738	down
HMDB0002304	379.27144	7.46	pos	leukotriene b4 ethanolamide		1.4938353	3.93963E-07	0.100497284	down
HMDB0003450	152.1197	7.645	pos	(-)-trans-carveol	C00964	1.924737266	0.00037816	2.426705443	up
HMDB0000315	332.23456	9.051	pos	16-alpha-hydroxypregnenolone	C06390	1.772079808	0.002316791	0.411939283	down
HMDB0002088	325.29734	9.513	pos	n-(2-hydroxyethyl)-9-octadecenamide		1.903295467	0.001426773	0.314349114	down

Table S7 DEMs discriminating LF21D from CK21D.

Compound ID	m/z	Retention time (min)	Ion mode	Metabolites	kegg	VIP	P-value	ratio	regulated
HMDB0000402	176.06804	3.626	neg	2-isopropylmalic acid	C02504	3.34159982	1.09E-06	0.407642535	down
HMDB0003265	610.1898	4.393	neg	hesperidin	C09755	2.645156037	7.17E-10	0.037554577	down
HMDB0002127	122.00342	5.254	neg	3-mercaptolactate	C05823	1.16073734	0.005304423	4.50956028	up
HMDB0033244	278.15141	7.816	neg	dibutyl phthalate	C14214	2.17337116	2.17E-12	0.041995036	down
HMDB0001552	145.03723	0.926	pos	2-oxoglutaramate	C00940	2.894753263	3.88E-05	0.419150309	down
HMDB0003543	72.05741	2.24	pos	butanal	C01412	3.103629653	8.76E-09	3.204877248	up
HMDB0004231	205.13103	2.849	pos	pantothenol	C05944	2.701072438	9.92E-12	30.43783314	up
HMDB0033115	143.07328	3.524	pos	6-methylquinoline		2.976634089	0.000304821	0.384909901	down
HMDB0062769	113.08384	3.59	pos	caprolactam	C06593	3.179780102	4.24E-09	3.591250473	up
HMDB0041140	248.10429	4.789	pos	3-methyl-2-butenyl caffeate	C10487	2.242878685	4.96E-05	2.220788731	up

HMDB0041802	143.07337	4.882	pos	naphthalen-2-amine	C02227	2.492982979	8.61E-06	0.169568682	down
HMDB0035593	222.16153	4.897	pos	rishitin	C09715	2.958169049	1.17E-05	25.66650872	up
HMDB0014855	298.19269	4.962	pos	norethindrone	C05028	2.614713743	0.000324236	0.155666054	down
HMDB0036688	232.14583	5.016	pos	costunolide	C09382	2.571829531	3.68E-06	9.247028007	up
HMDB0033244	278.15125	5.016	pos	dibutyl phthalate	C14214	2.38043277	2.87E-07	14.30057267	up
HMDB0005102	336.22934	5.219	pos	prostaglandin d1	C06438	2.778689706	2.28E-07	0.019054689	down
HMDB0035695	282.19795	5.233	pos	dehydroretinaldehyde	C05918	1.96410832	0.000195145	0.246964521	down
HMDB0014852	267.12538	5.652	pos	apomorphine		3.006153602	2.67E-06	0.035041039	down
HMDB0010217	318.21887	5.661	pos	5-oxoete	C14732	2.641343794	1.13E-06	0.108889506	down
HMDB0002304	379.2715	5.746	pos	leukotriene b4 ethanolamide		2.570397843	2.45E-07	0.006674645	down
HMDB0035593	222.16149	5.753	pos	rishitin	C09715	2.933766761	6.69E-07	13.59073667	up
HMDB0035695	282.19795	5.797	pos	dehydroretinaldehyde	C05918	2.074227005	0.001093321	0.195229807	down
HMDB0033244	278.15126	5.891	pos	dibutyl phthalate	C14214	2.241295302	1.40E-05	6.080686054	up
HMDB0033115	143.07331	6.043	pos	6-methylquinoline		2.032371366	0.000310301	0.209986915	down
HMDB0013629	395.26619	6.089	pos	prostaglandin d2 ethanolamide		2.530533984	9.62E-05	12.96709381	up
HMDB0059925	180.1146	6.475	pos	2-tert-butyl-4-methoxyphenol		1.489725612	7.01E-07	2.578851087	up
HMDB0061861	290.18751	6.653	pos	octyl methoxycinnamate		3.062181352	4.92E-07	4.418175258	up
HMDB0014604	228.11444	6.948	pos	nabumetone		3.347999521	3.54E-06	33.3291769	up
HMDB0015603	473.2652	8.698	pos	ximelagatran		2.979870547	8.36E-06	0.471827496	down
HMDB0015432	557.42864	9.759	pos	vecuronium	C07553	1.756872534	0.003007874	3.171717066	up

Supplementary figure

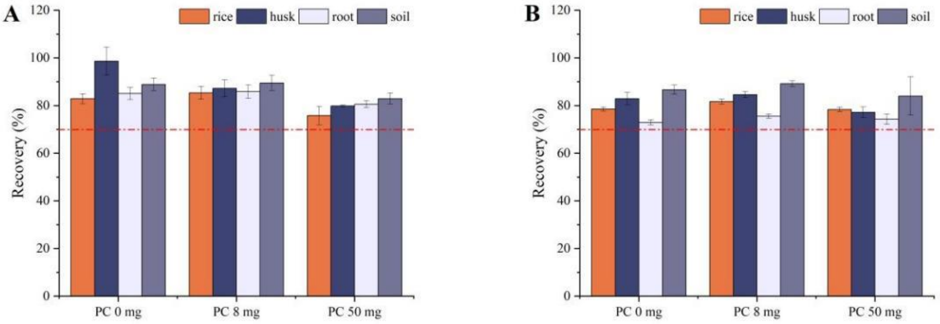

Figure S1 Effect of purification materials (PSA 50mg + C_{18} 50mg + $MgSO_4$ 150mg) containing varying contents of PC for FXP (A) and MFZ (B) in different matrices at the 0.01 mg/kg level (n = 3).

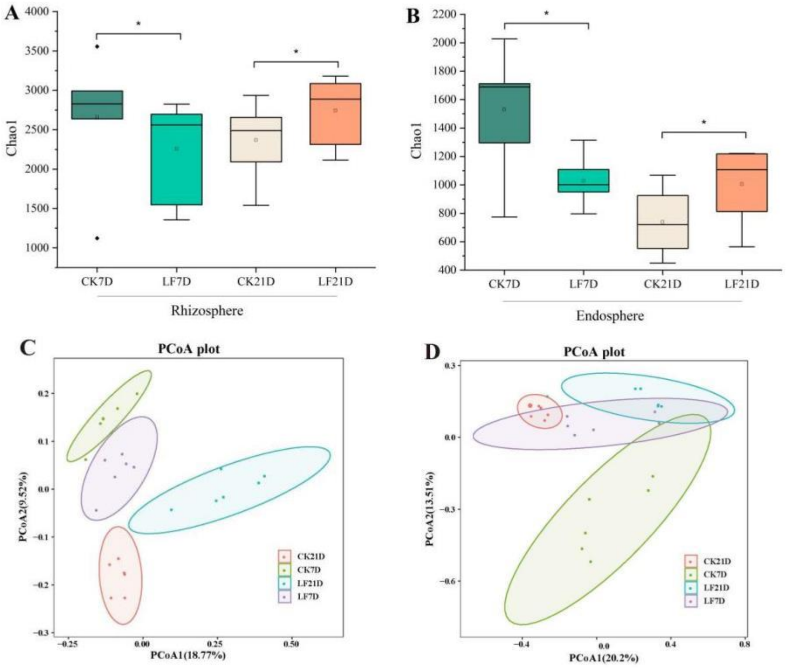

Figure S2 Alpha and beta diversities of root-associated bacterial communities at S and T treatments. Chao1 indexes of bacterial communities among soil-root samples between the rhizosphere (A) and endophytes (B). PCoA analysis of bacterial communities among among soil-root samples between the rhizosphere (C) and endophytes (D).

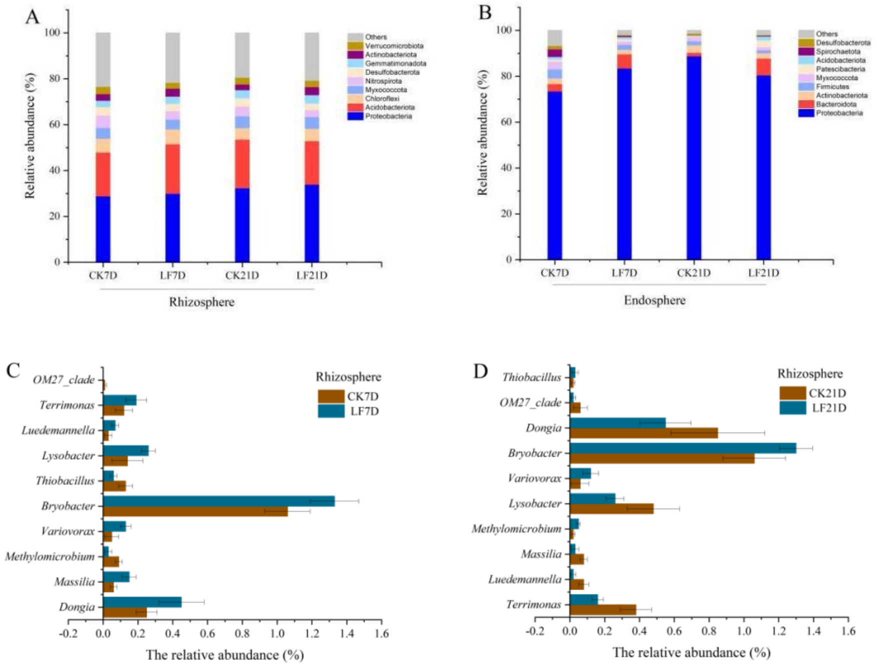

Figure S3 The relative abundances of the major bacteria in CK7D, LF7D, CK21D and LF21D at the phylum level (A and B). The relative abundance of bacterial genera with significant differences in the rhizosphere under S (C) and T (D) treatments.

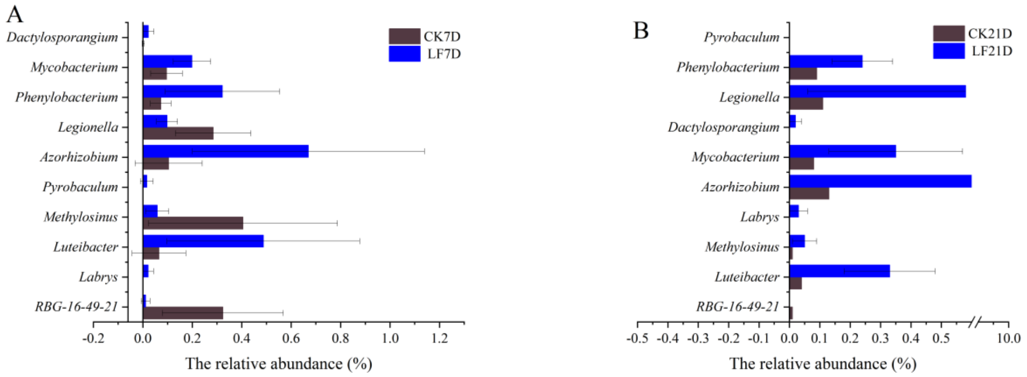

Figure S4 The relative abundance of bacterial genera with significant differences in endosphere under CK7D/LF7D (A) and CK21D/LF21D (B) treatments.

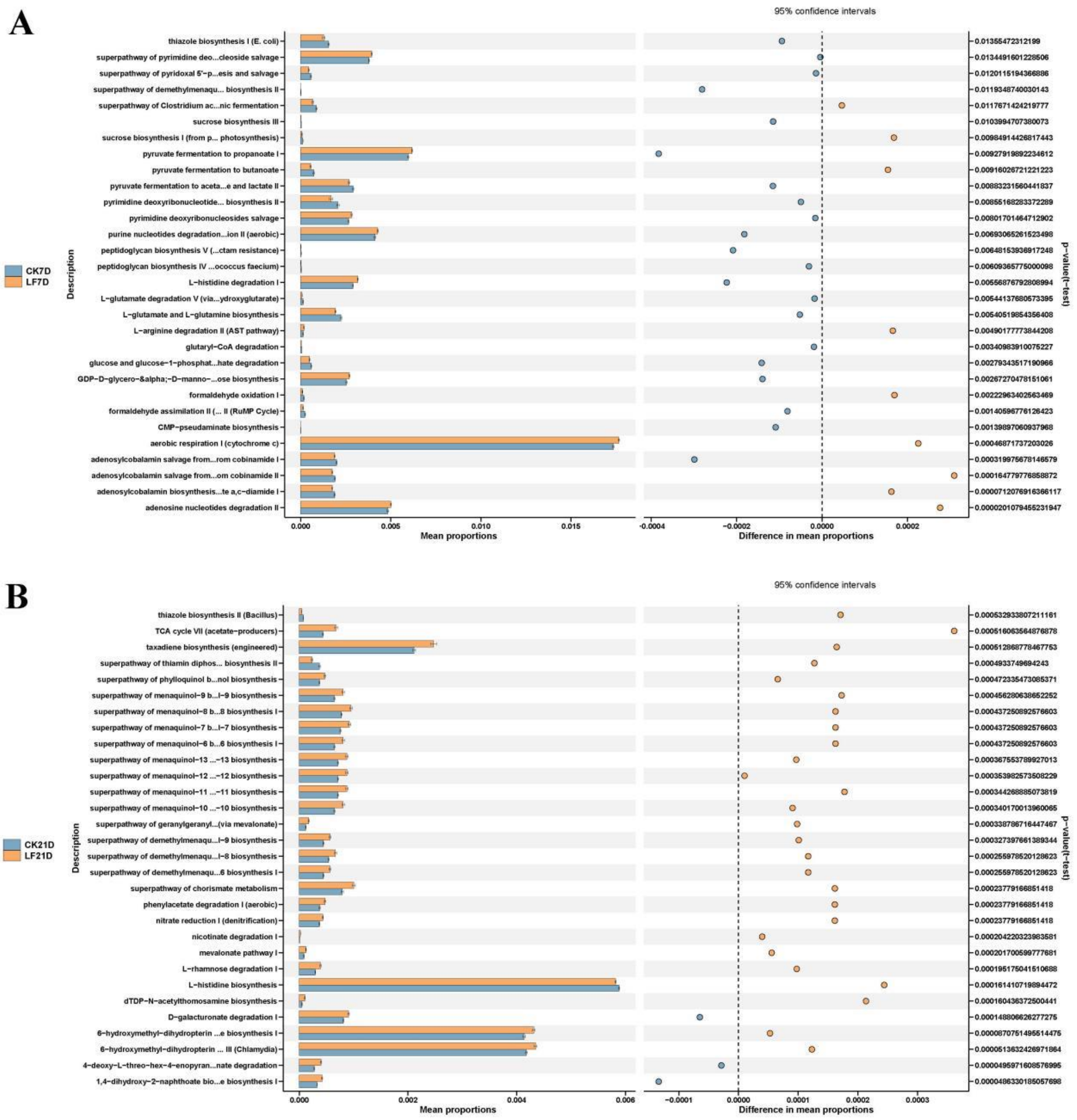

Figure S5 Functional prediction of KEGG pathways in microbial communities among the LF7D/CK7D (A) and LF21D/CK21D (B) treatments using STAMP difference analysis.

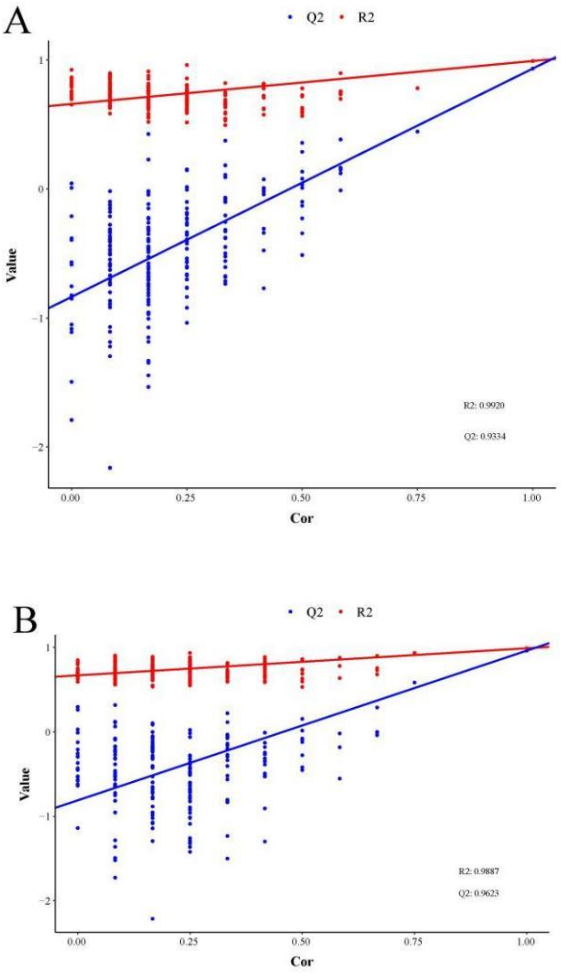

Figure S6 Model validation diagram of partial least squares-discriminant analysis (PLS-DA) of metabolites extracted from rice samples after spraying mefentrifluconazole and fluxapyroxad at LF7D/CK7D (A) and LF21D/CK21D treatments (B).

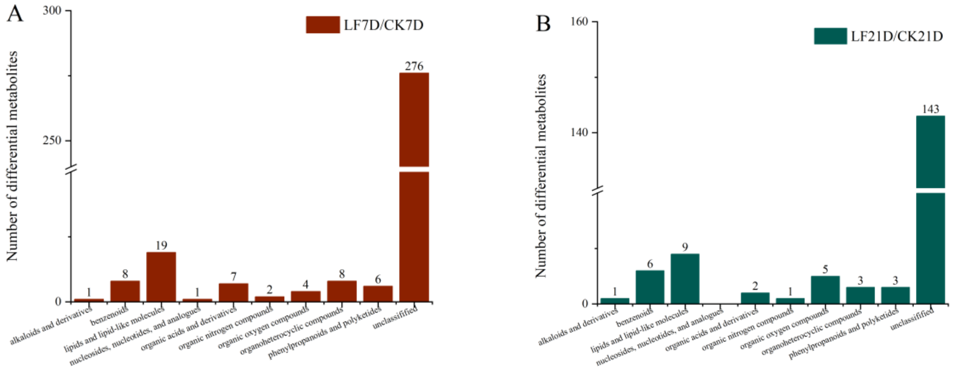

Figure S7 The classifification of differential metabolites in CK7D/LF7D (A) and CK21D/LF21D (B) treatments.

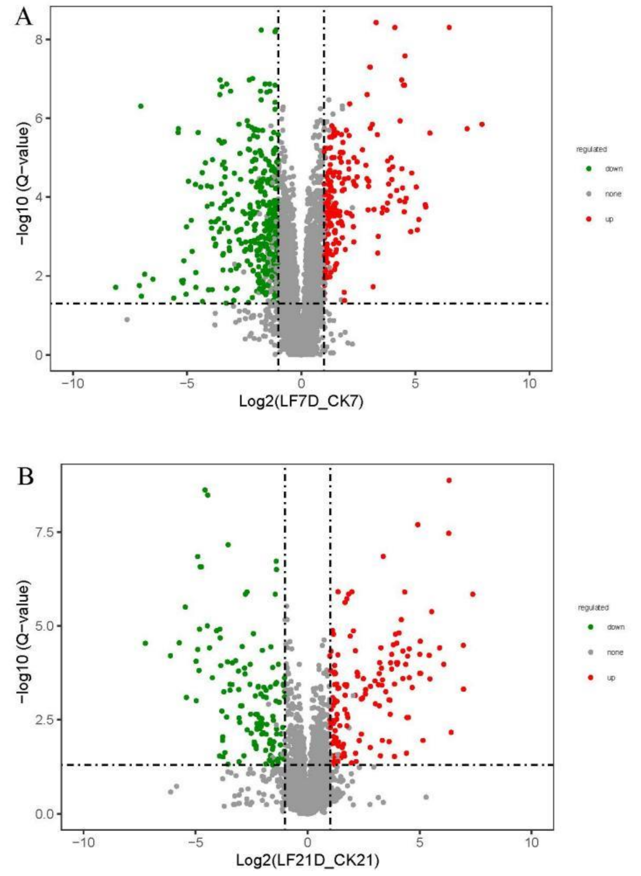

Figure S8 Volcano plots of differential metabolites in CK7D/LF7D (A) and CK21D/LF21D (B) treatments.

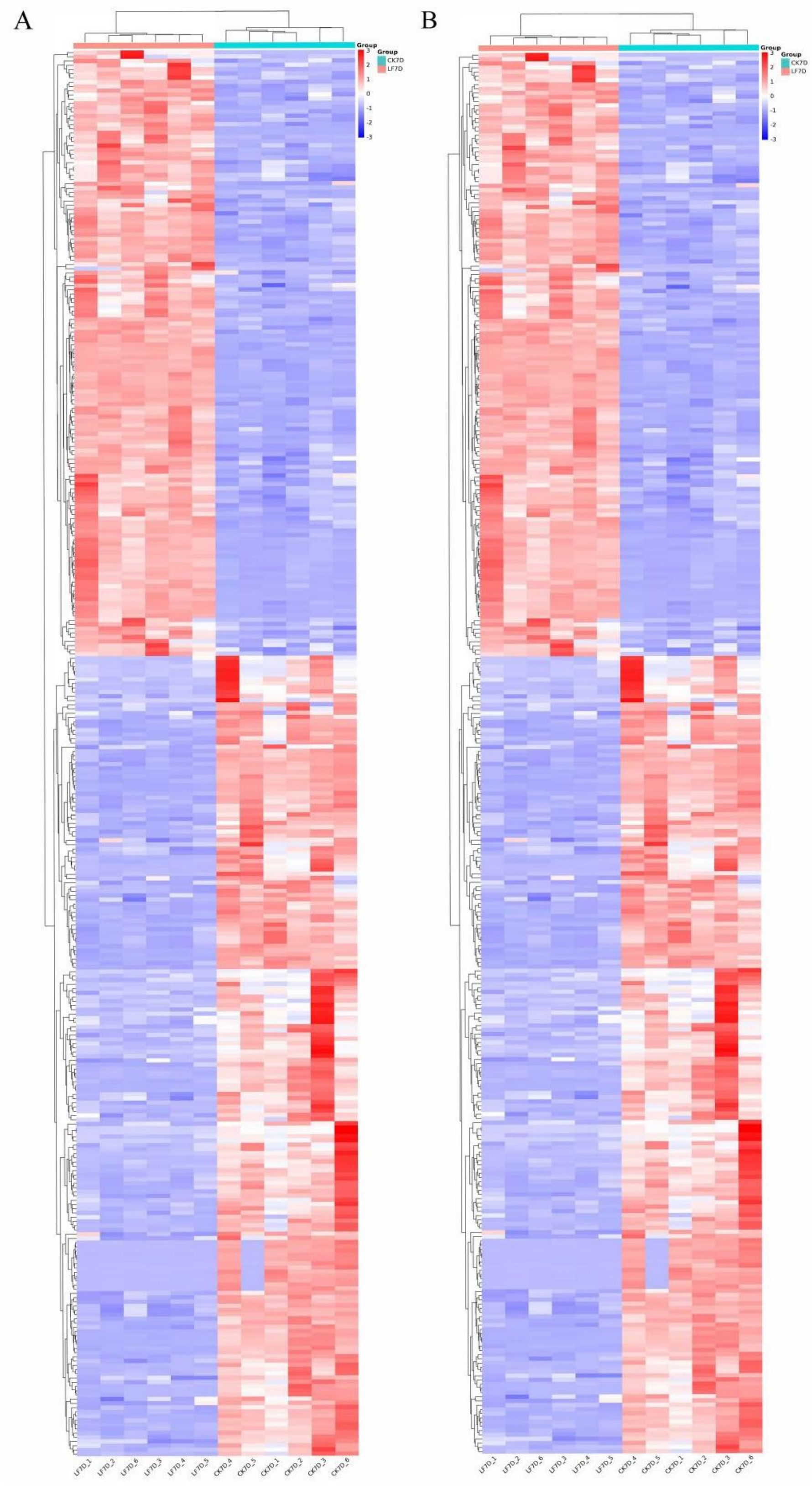

Figure S9 Hierarchical clustering analysis of differential metabolites in CK7D/LF7D (A) and CK21D/LF21D (B) treatments.

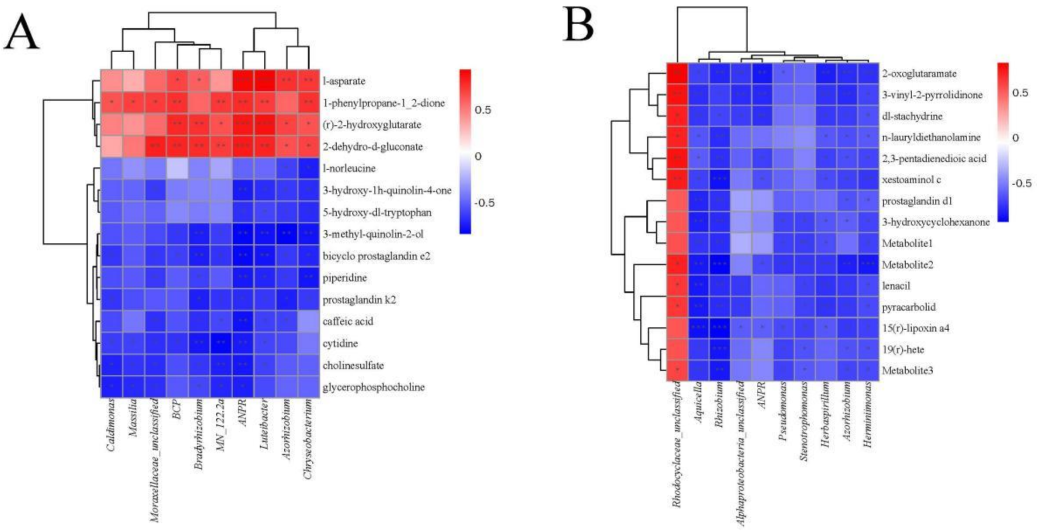

Figure S10 Heat map of the correlation between endophytes and differential metabolites among the LF7D/CK7D (A) and LF21D/CK21D (B) treatments. The red and blue indicate positive and negative correlations. The color depth indicate the degree of correlation (*BCP: burkholderia-caballeronia-paraburkholderia*. *ANPR:allorhizobium-neorhizobium-pararhizobium-rhizobium*. Metabolite 1: (1s_4s)-4-hydroxy-3-oxocyclohexane-1-carboxylate. Metabolite 2: 15-deoxy-12,14-prostaglandin d2. Metabolite 3:15-epi prostaglandin a1.$^{*}P$<0.05, $^{**}P$<0.01, $^{***}P$<0.001).
